# Identification of histopathological classification and establishment of prognostic indicators of gastric adenocarcinoma based on deep learning algorithm

**DOI:** 10.1007/s00795-024-00399-8

**Published:** 2024-08-01

**Authors:** Zhihui Wang, Hui Peng, Jie Wan, Anping Song

**Affiliations:** 1 Department of Ultrasound Imaging, Tongji Hospital, Tongji Medical College, Huazhong University of Science and Technology, Wuhan, 430101 Hubei China; 2 Department of Oncology, Tongji Hospital, Tongji Medical College, Huazhong University of Science and Technology, Wuhan, 430101 Hubei China; 3 Department of Pathology, Tongji Hospital, Tongji Medical College, Huazhong University of Science and Technology, Wuhan, 430101 Hubei China; 4 Department of Oncology, Tongji Hospital Sino-French New City Branch, Caidian District, No.288 Xintian Avenue, Wuhan, 430101 Hubei China

**Keywords:** Gastric adenocarcinoma, Subtype, Deep learning, Whole-slide images

## Abstract

The aim of this study is to establish a deep learning (DL) model to predict the pathological type of gastric adenocarcinoma cancer based on whole-slide images(WSIs). We downloaded 356 histopathological images of gastric adenocarcinoma (STAD) patients from The Cancer Genome Atlas database and randomly divided them into the training set, validation set and test set (8:1:1). Additionally, 80 H&E-stained WSIs of STAD were collected for external validation. The CLAM tool was used to cut the WSIs and further construct the model by DL algorithm, achieving an accuracy of over 90% in identifying and predicting histopathological subtypes. External validation results demonstrated the model had a certain generalization ability. Moreover, DL features were extracted from the model to further investigate the differences in immune infiltration and patient prognosis between the two subtypes. The DL model can accurately predict the pathological classification of STAD patients, and provide certain reference value for clinical diagnosis. The nomogram combining DL-signature, gene-signature and clinical features can be used as a prognostic classifier for clinical decision-making and treatment.

**Supplementary Information:**

The online version contains supplementary material available at 10.1007/s00795-024-00399-8.

## Introduction

Gastric adenocarcinoma (STAD) accounts for more than 95% of all gastric malignancies and is the most common cause of cancer-related death [1, 2]. According to the Global Cancer Statistics Report 2020, there are about 1.07 million new cases of STAD, accounting for 5.6% of all new cancer cases and ranking fifth in terms of incidence rate, and about 769,000 deaths, accounting for 7.7% of all cancer deaths and ranking fourth in terms of mortality rate [3]. Patients with early gastric cancer undergoing radical surgery and subsequent chemotherapy have a 5-year survival rate of 90% after surgery [4]. However, more than half of STAD patients are initially diagnosed at an advanced stage, and the 5-year overall survival (OS) rate of STAD is less than 30% [5]. Tumor microenvironment (TME) denotes the non-cancerous cells and components presented in the tumor, including molecules produced and released by them. As an important component of TME, tumor-infiltrating immune cells (TIIC) are associated with the promotion or inhibition of tumor growth [6, 7]. Therefore, early detection and appropriate treatment are important ways to reduce the mortality of STAD patients, while understanding the degree of immune cell infiltration in different subtypes of gastric cancer is helpful for the administration of relevant immunotherapy.

The spatial characteristics of different tissues in histopathological images play an important role in the diagnosis and prognosis of cancer [8–10]. Traditionally, pathologists have identified and distinguished different pathological types of STAD by visual examination of hematoxylin and eosin(H&E)-stained histopathologic sections. However, this method is labor-intensive, tedious, and time-consuming, and the diagnostic accuracy is negatively affected by the acute shortage of pathologists and heavy diagnostic workloads [11]. To overcome these limitations, researchers have turned to deep learning (DL) based approaches that harness the power of artificial intelligence and neural networks to automate and enhance the analysis of histopathology images [12–14]. CLAM (Clustering-constrained Attention Multiple Instance Learning) is a deep-learning-based weakly supervised method that uses attention-based learning to automatically identify subregions of high diagnostic value to accurately classify the whole slide, while also enabling the use of instance-level clustering over the representative regions identified to constrain and refine the feature space [15]. In practical applications, CLAM has shown superior performance, particularly in medical image analysis. For instance, in cancer detection and classification tasks on pathology images, CLAM accurately identifies cancerous regions by incorporating clustering constraints, significantly enhancing diagnostic accuracy and efficiency [16, 17].

Recently, a DL method has been developed based on histopathological images, which has shown great potential for the rapid detection of adenocarcinoma in gastric biopsy and resection specimens. It exhibits high sensitivity and specificity and is beneficial for future diagnostic pathology workflows. In addition, it enables accurate segmentation of STAD regions, enabling further analysis and supporting translational research [18, 19]. Studies have shown that DL algorithms applied to H&E stained slides can predict microsatellite instability in STAD as well as specific mutations in other cancers [20, 21]. Osamu Iizuka et al. used convolutional neural networks (CNN) and recurrent neural networks (RNNs) to classify the biopsy histopathology WSIs of the stomach with an accuracy of more than 90% [22]. Huang et al. designed a CNN-based model, Gastro-MIL, for accurate diagnosis of STAD directly from digital H&E-stained images. The model has an accuracy of 92%, which is comparable to the discrimination ability of professional pathologists [23]. These DL methods have brought great hope to improve the accuracy and efficiency of STAD diagnosis and classification. By automated analysis of H&E-stained histopathological images, interobserver variability can be effectively reduced and more objective and reproducible results can be provided. In addition, it may be possible to reveal novel features of specific pathological subtypes, thereby contributing to the development of more targeted therapeutic strategies.

In this study, we created a DL model to classify adenocarcinomas and mucinous adenocarcinomas from histopathological images to support conventional histopathology diagnosis by expert pathologists. The TCGA-STAD cohort was used for training, followed by validation using an external independent dataset to further illustrate the generalization ability of the model. In addition, STAD patients were successfully classified into two subtypes with different molecular characteristics based on DL features combined with transcriptome datasets, which further explored the pathogenic mechanism at the genome scale.

## Materials and methods

### Patient cohorts

The 356 STAD patients with clinical characteristics and mRNA sequencing data were acquired from The Cancer Genome Atlas (TCGA) (https://portal.gdc.cancer.gov/). 356 WSIs of STAD were obtained from the TCGA-STAD cohort, including 322 cases of adenocarcinoma and 34 cases of mucinous adenocarcinoma (Scanned slides with extensive labeling in the area of the covered tissue, damaged slides, and slides that did not contain tumors were excluded, and only one sample was selected per patient.). In addition, 80 H&E-stained WSIs of STAD were obtained from Shanghai Zhuoli Biotech Company (Shanghai, China) and used for external validation. The ethical approval of validation cohort was obtained from the Tongxu County People's Hospital, Henan, China. The TCGA database is publicly available for research and therefore does not require ethical approval.

### DL feature extraction and selection

Considering the very large image size of WSIs (typically 100,000 * 80,000 pixels), WSIs were cropped into many patches. Tissue regions were then exhaustively split into patches of 256 × 256 pixels (without overlapping) at 20 × using the OpenSlide library in Python. Feature vectors were extracted using a modified ResNet50 model pre-trained on ImageNet, by feeding it with a cropped pixel size of 256 × 256 patches. Finally, each patch was output as a 1024-dimensional feature vector using adaptive averaging of spatial pools after selecting the third residual block in the ResNet50 model.

### DL models

356 WSIs of STAD were randomly divided into a training set (80%), validation set (10%) and test set (10%) for DL via clam as a way to construct pathohistological typing of STAD, and further estimated it robustness in the external validation set. During the training process, weakly supervised learning is employed, where each WSI is assigned a slide-level label indicating whether it belongs to adenocarcinoma or mucinous adenocarcinoma. Throughout training and inference, the model utilizes an attention-based pooling function to aggregate patch-level features into slide-level representations for classification. The model examines and ranks each patch within the tissue regions of the WSI, assigning an attention score to each patch, which reflects its contribution or importance to the collective slide-level classification of a specific category. By leveraging attention-based learning, the model can identify and aggregate regions of high diagnostic significance, thereby providing a slide-level classification for each WSI. The training was performed using a tenfold Monte Carlo cross-validation strategy. The training was performed using a tenfold Monte Carlo cross-validation strategy. Performance was further assessed using the area under the curve (AUC) from a receiver operating characteristic curve (ROC).

### Attention map generation

CLAM is capable of generating interpretable heat maps that enable an intuitive analysis of the relative contribution of each tissue region to model predictions in each WSI [15]. These heat maps provide pathologists with insights into histological and cytological features that are strongly associated with high predictive value. To account for the relative importance of different regions in the pathological picture for the model's final level predictions, we calculated and saved unstandardized attention scores for all patches extracted from the pathological picture using the attention branches corresponding to the model's predicted categories. The attention score was learned by CLAM for each patch and converted into percentiles. For each WSI, the percentiles were then normalized to [0, 1] with 1 being the most predictive and 0 being the most non-informative. The normalized scores were converted to RGB colors using heat maps and displayed above their respective spatial locations in the pathology pictures to visually identify and interpret areas of high attention displayed in red and areas of low attention displayed in blue.

### Unsupervised cluster of DL features

The least absolute shrinkage and selection operator (LASSO) analysis was used to select the most useful DL features among 1024 features, and the optimal values of the penalty parameter λ were determined by tenfold cross-validations. The importance of each feature was evaluated by the weight coefficient of DL. The larger the parameter estimate (absolute value), the higher the importance of the element. To gain more insight into the molecular mechanism of STAD, we performed an unsupervised clustering analysis to identify subgroups with similar patterns based on DL features, using the kmeans algorithm in the R package "Consensus Cluster Plus". Kaplan–Meier (K-M) curves were used to compare the prognosis of subgroups defined by DL features.

### Transcriptome analysis of different histopathological subtypes

#### Identification of differentially expressed genes (DEGs)

The “limma” package in R software was utilized to screen DEGs of different histopathological subtypes. An adjusted p value of < 0.05 and log2 |Fold Change|> 0.5 were considered statistically significant. The “ggplot2” package in R was used to plot the volcano map in the two groups. The Significant DEGs were further screened by LASSO regression analysis.

#### Functional enrichment analysis

Based on the Gene Ontology (GO) database and Kyoto Encyclopedia of Genes and Genomes (KEGG) Pathway database, the “clusterProfiler” R package was used to perform functional enrichment analysis of the DEGs according to the pathological subtypes. The p-value of less than 0.05 was identified as a significant term.

#### Estimation of the immune cell infiltration

Immune-related gene set were obtained from Genecard database (Supplementary Table S1) and intersected with DEGs. The single-sample gene set enrichment analysis (ssGSEA) was used to estimate the relative abundance of different immune cell types in each sample. Then the correlation between the scores of each immune cell was calculated, and the differences in immune scores and immune checkpoint genes between subtypes were tested by Wilcoxon test.

#### Identification of histopathologic DL-signature and gene‑signature

The “limma” R package was used to screen the DEGs between adenocarcinoma and mucinous adenocarcinoma in the TCGA-STAD cohort, and the intersection of the immune-related DEGs after pathological prediction and classification was used to select the candidate gene signature. Multivariate Cox modeling was used to create gene signatures, and a risk score was determined by a linear combination of the regression coefficient (α) from the multivariate Cox regression model and gene expression levels based on the "ggrisk" package. According to the median risk score, all patients were divided into a high-risk group and a low-risk group, and the K-M survival curve was drawn to compare the survival rate of the two groups. Pathological features were derived from the DL features identified by the LASSO penalty model. Based on DL characteristics, genetic characteristics and clinical characteristics, a nomogram was constructed to predict OS.

### Statistical analysis

All analyses were performed with R (version 4.3.1) or Python (version 3.7.12). The versions of the Python libraries and R packages used are in Supplementary Table S2. The Wilcoxon test was used to analyze the differences between the two groups. Correlations between variables were determined using Pearson's analysis. Survival analysis was conducted using the “survival” R package, and the log-rank test was performed with the “survdiff” function. All statistical tests were considered significant with *p* < 0.05.

## Results

### Performance of the histopathological classifier

**Fig. 1:**
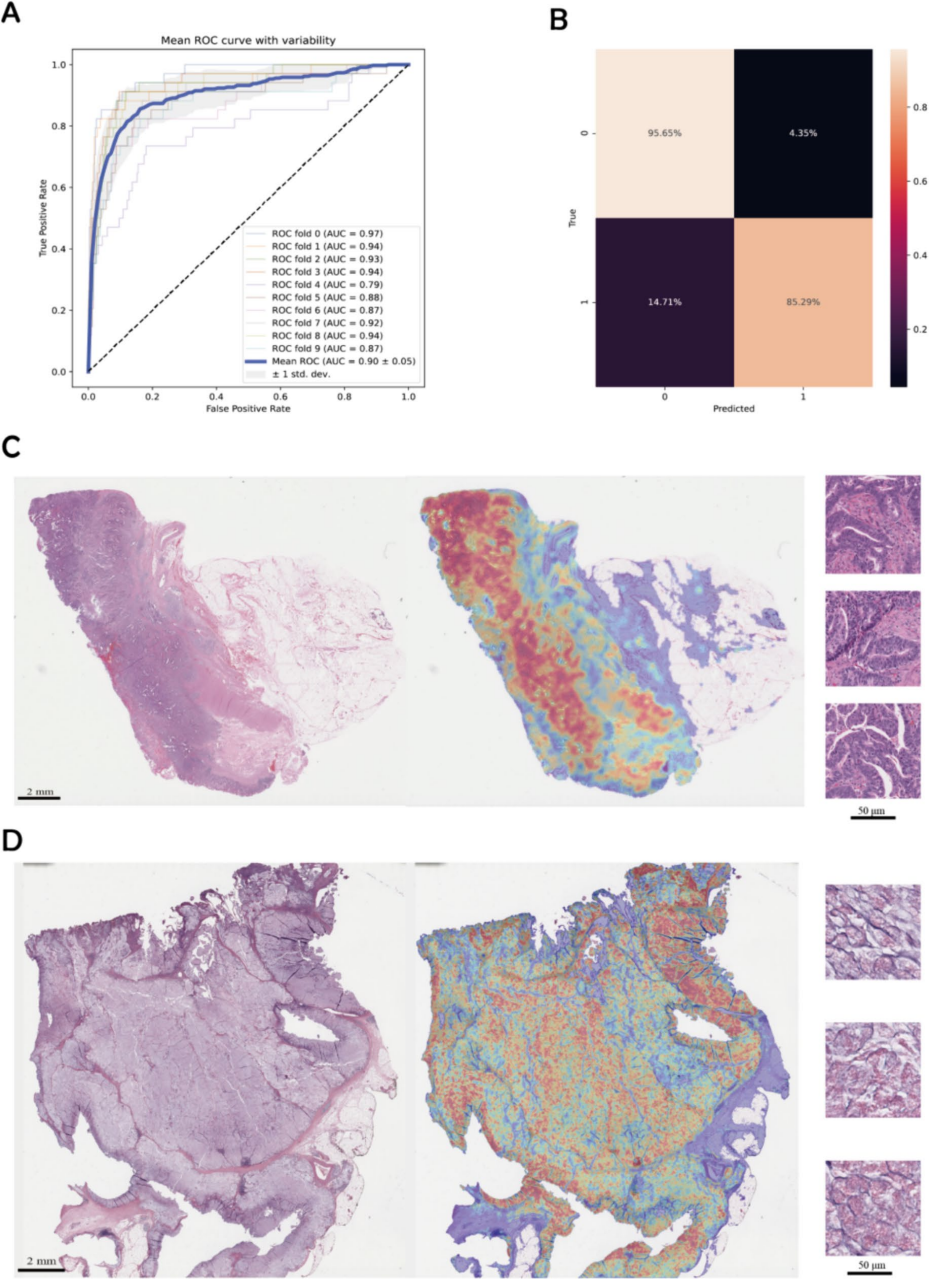
DL algorithm predicts pathologic staging in STAD patients. **A** The ROC curve and AUC value of DL model in TCGA-STAD cohort. **B** The confusion matrix of DL model. **C** Attention heat map of pathological tissue sections of adenocarcinoma. **D** Attention heat map of pathological tissue sections of mucinous adenocarcinoma. Areas of high attention are shown in red and areas of low attention are shown in blue

A pathology-based DL model was developed in the training set of the TCGA-STAD cohort (8:1:1 for training, validation and testing). The tenfold Monte Carlo cross-validation was used to evaluate the classification performance of CLAM in clinical diagnostic tasks. The results showed that each model had good performance in recognizing adenocarcinoma and mucinous adenocarcinoma, with an average AUC of 0.90 (maximum 0.97, minimum 0.78) (Fig. 1A). Further confusion matrix results show that the correct rate of adenocarcinoma recognition is 95.65% and the success rate of mucinous adenocarcinoma recognition is 85.29%, both of which reflect high recognition rates (Fig. 1B). To visualize and interpret the relative importance of each region in the WSIs, we converted the attention scores of the model's predicted categories to percentiles, normalized them and mapped them to the original slides to generate an attention heatmap. Two representative heatmaps providing patch-level predictions for adenocarcinoma and mucinous carcinoma, respectively, are shown in Fig. 1C-D.

### External validation model

**Fig. 2:**
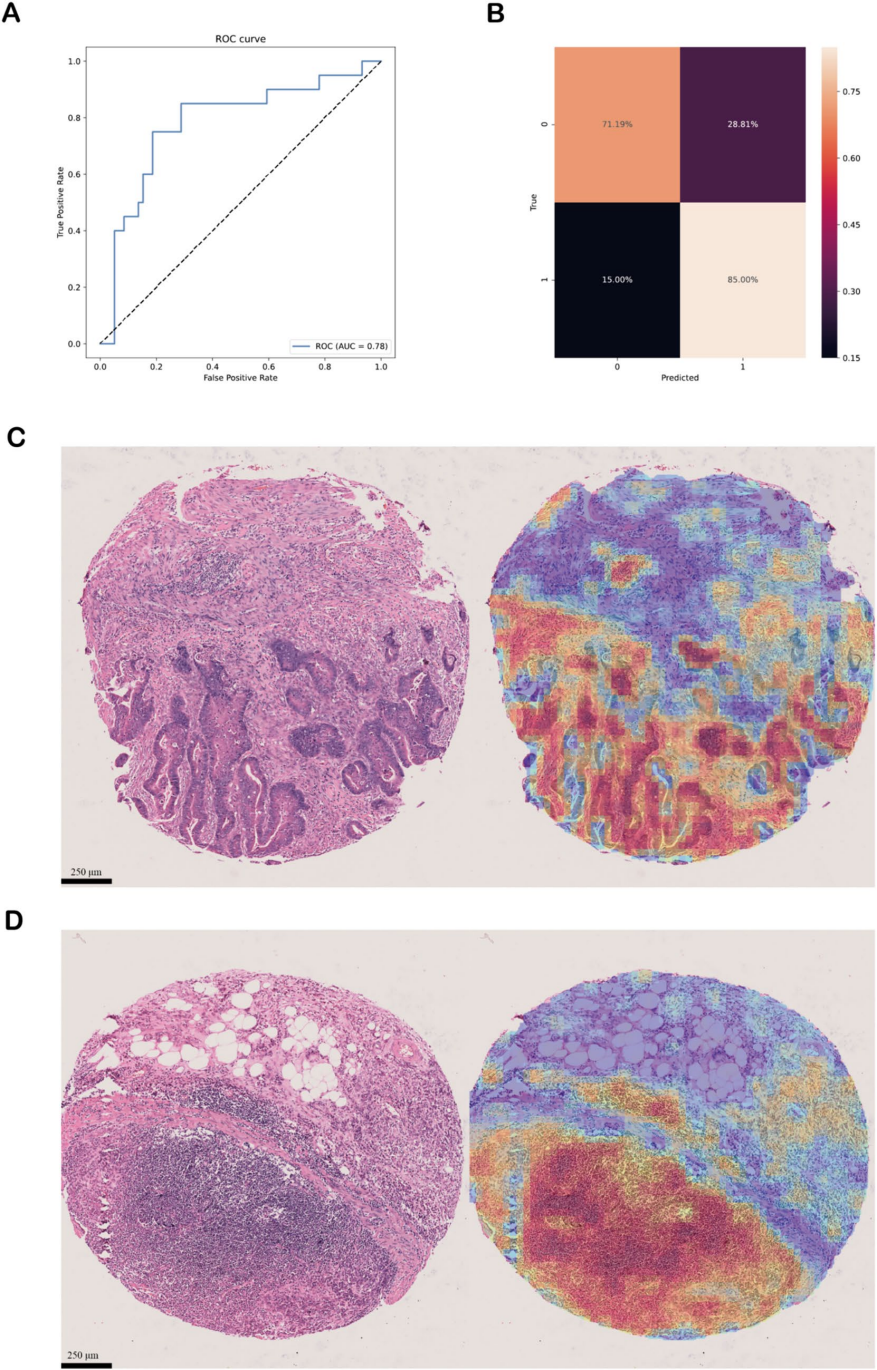
**A** The ROC curve and AUC value of DL model in validation cohort. **B** The confusion matrix of DL model in validation cohort. **C** Attention heat map of pathological tissue sections of adenocarcinoma in validation cohort. **D** Attention heat map of pathological tissue sections of mucinous adenocarcinoma in validation cohort. Areas of high attention are shown in red and areas of low attention are shown in blue

DL systems are prone to overfitting the data they are trained on, so we introduced H&E-stained section data of 80 STAD cases (59 adenocarcinomas, 21 mucinous adenocarcinomas) for external validation. It was worth noting that the external validation dataset and the TCGA-STAD cohort are vastly different in terms of both patient ethnicity and slice preparation techniques. Considering these, we used an external cohort for validation and achieved an AUC of 0.78 in the dataset, suggesting that the DL model has good generalization capabilities(Fig. 2A). The results of the confusion matrix showed that adenocarcinoma was identified with a success rate of 71.19% and mucinous adenocarcinoma was identified with a success rate of 85% (Fig. 2B). In addition, to further validate the reliability of the analysis results, we invited pathologists to review the attention heat maps identified by CLAM. The attentional heat maps of pathological tissue sections of adenocarcinomas and mucinous adenocarcinomas in the validation cohort are shown in Fig. 2C-D, with the red areas corresponding to tumour regions.

### Unsupervised cluster of DL features

**Fig. 3:**
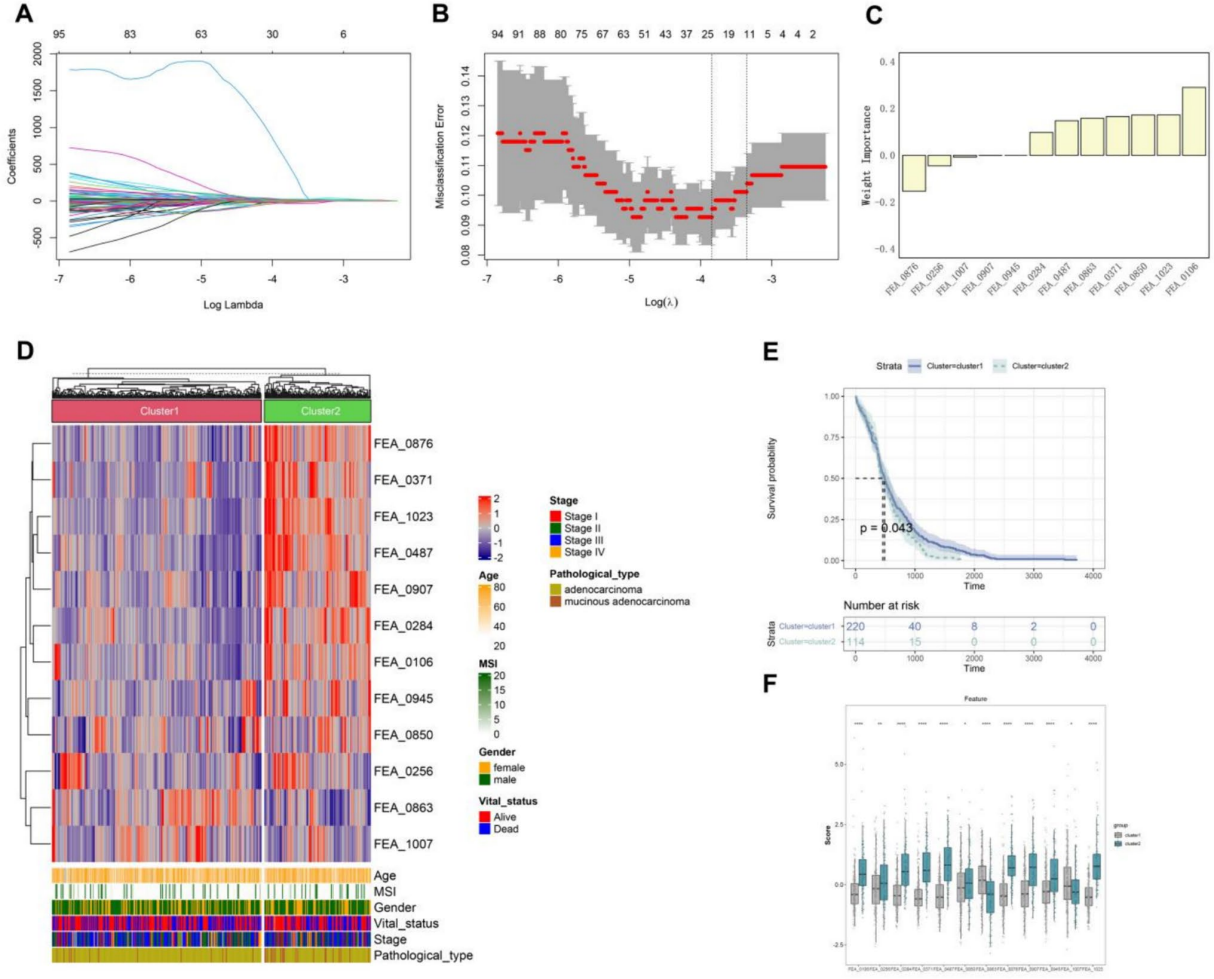
Selection of DL features and unsupervised clustering. **A** LASSO coefficient profiles of all DL features. **B** Cross-validation to select the optimal tuning parameter log (λ) in LASSO regression analysis. **C** Bar plot of DL feature weights. **D** Comprehensive heatmap of unsupervised clustering of DL features combined with clinical features. **E** K-M curve of survival probability between Cluster 1 and Cluster 2 subtypes. **F** Boxplots of distribution differences between cluster 1 and cluster 2 for the 12 DL features

Using 1024 DL-features, we identified 12 features by the LASSO-penalted feature selection, and the relative importance of the features is shown in the figure (Fig. 3A-C). The 12 DL features were further conducted to investigate the key clusters in the TCGA-STAD cohort. Using unsupervised clustering (*k* = 2), two stable subtypes were able to be identified: cluster 1 (236 STAD patients) and cluster 2 (120 STAD patients). Clusters1 contained 223 cases of adenocarcinoma and 13 cases of mucinous adenocarcinoma, while cluster2 contained 99 cases of adenocarcinoma and 21 cases of mucinous adenocarcinoma. We then created a comprehensive heatmap to show associations between subtypes and clinical features. The results showed that patients with mucinous adenocarcinoma were mainly concentrated in cluster 2 (Fig. 3D). Meanwhile, the K-M curve showed that the survival probability of STAD patients in cluster 2 was lower than that in cluster 1 (*P* < 0.043) (Fig. 3E), suggesting that the prognosis of mucinous adenocarcinoma is worse. In addition, the boxplot results showed that the distribution of all 12 DL-features was significantly different between cluster 1 and cluster 2 (Fig. 3F).

### Functional enrichment analysis of DEGs in different histopathological subtypes

**Fig. 4:**
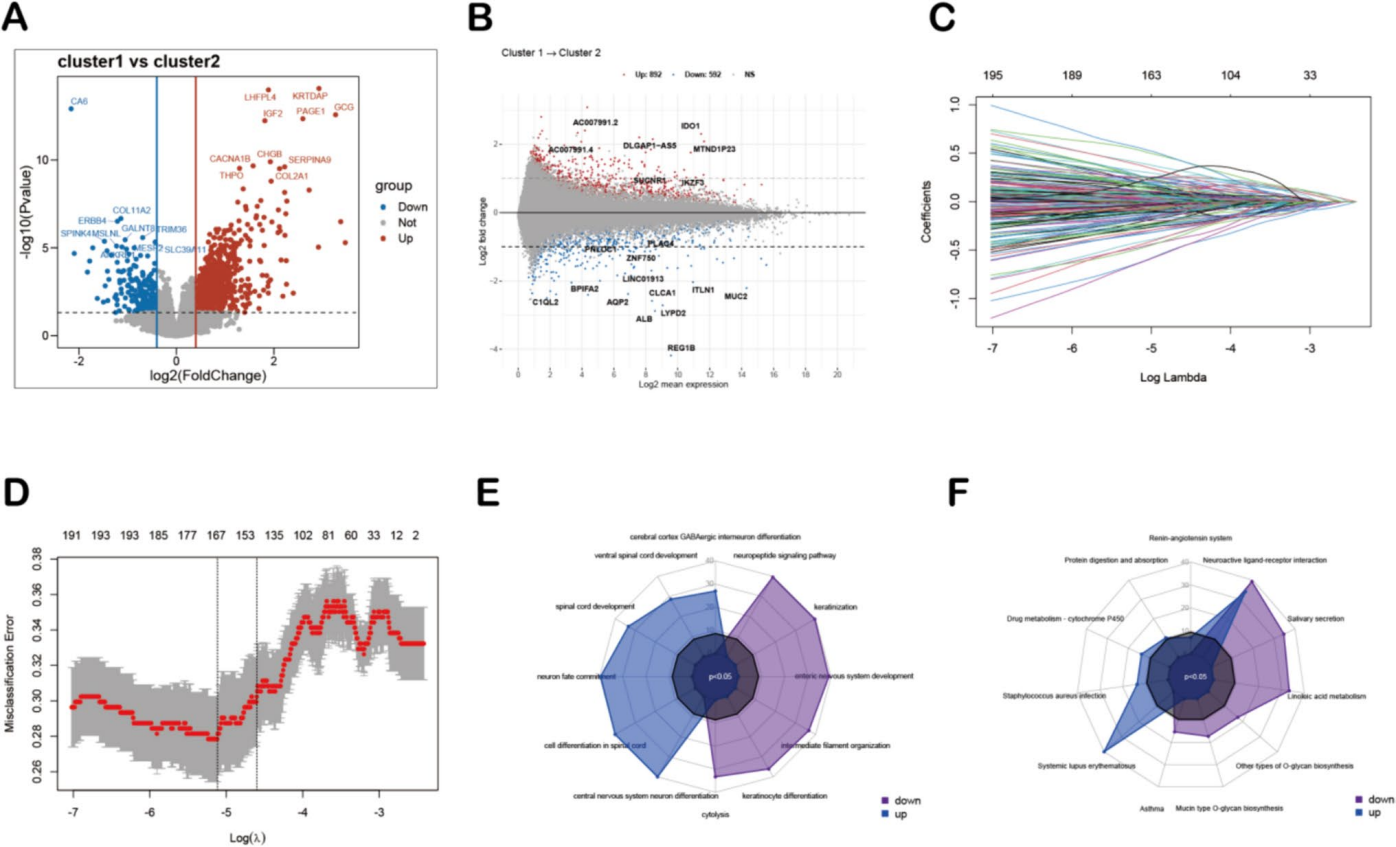
Transcriptome differential analysis between cluster 1 and cluster 2 subtypes. **A** Volcano plot of DEGs between cluster 1 and cluster 2 subtypes. The cut-off criteria were |log2FC|> 0.5 and *P* < 0.05. The red dots represent the up-regulated genes, and the blue dots denote the down-regulated genes. The grey dots indicate the genes with |log2Fc|< 0.5 and/or *P* > 0.05. **B** The MA plot of different subtypes. **C** LASSO coefficient profiles of DEGs. **D** The distribution of the lowest mean squared error with the corresponding penalization lambda value in LASSO-penalized model. **E** GO functional analysis showing enrichment of DEGs. **F** KEGG pathway enrichment analysis of DEGs

We screened 1287 DEGs between the cluster 1 and the cluster 2 in TCGA-STAD using the R package “limma” (*P* < 0.05, |log2FC|> 0.5) (Fig. 4A). 145 genes were further screened based on LASSO regression and tenfold cross-validation (Fig. 4B-C). GO analysis and KEGG analysis were conducted to obtain the biological functions of 145 DEGs to understand which signaling pathways might serve as an important role in STAD. The results showed that DEGs were mainly enriched in signaling pathways such as protein digestion and absorption and enteric nevous system development (Fig. 4D-E).

### Landscape of immune characteristics in histopathological subtypes

**Fig. 5:**
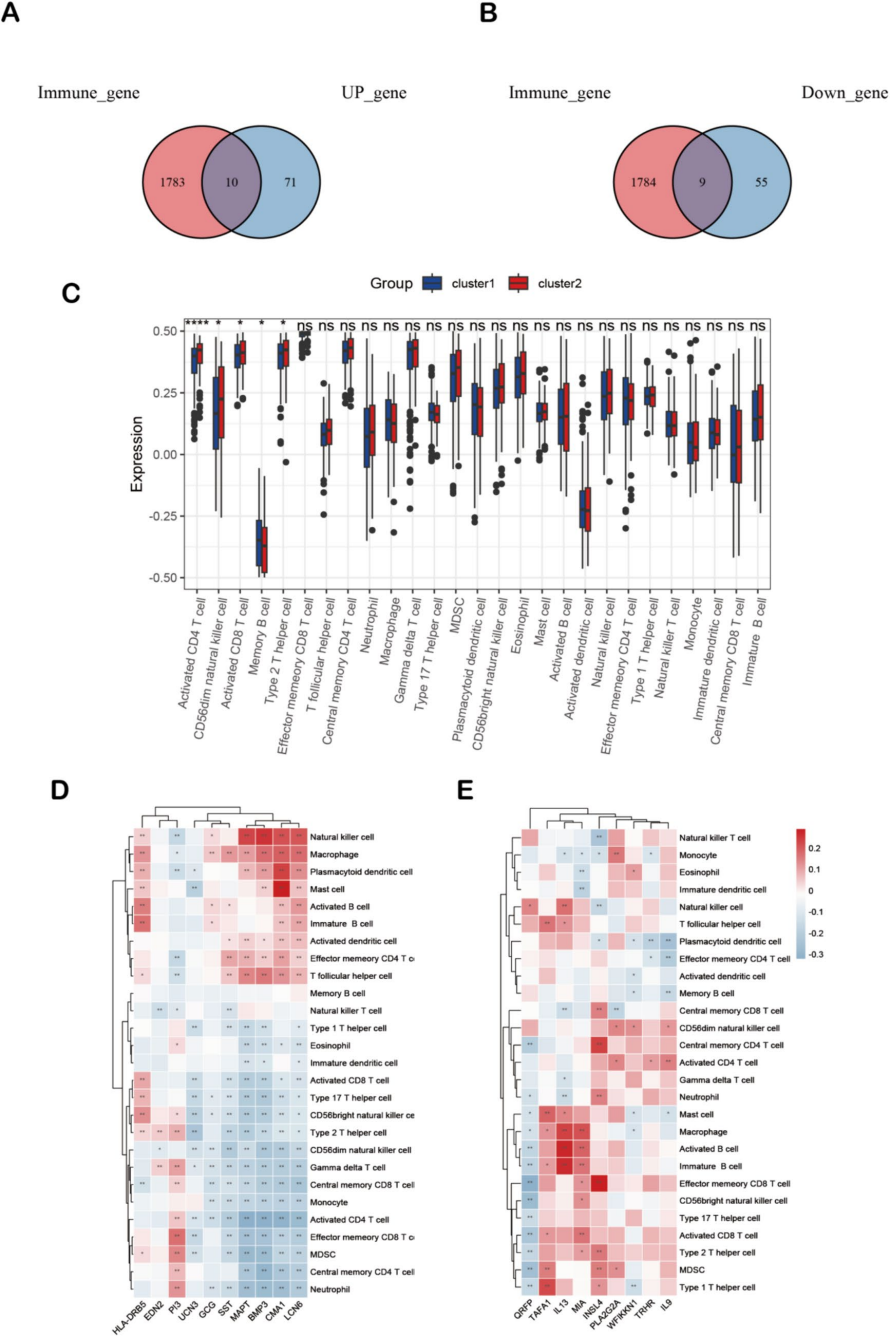
Immune infiltration analysis between cluster 1 and cluster 2 subtypes. **A** Venn diagram of the intersection of up-regulated genes and immune-related genes. **B** Venn diagram of the intersection of down-regulated genes and immune-related genes. **C** Boxplot of the distribution difference of immune cells between cluster 1 and cluster 2 subtypes. **D** Correlation between up-regulated gene expression and immune cell infiltration. **E** Correlation between down-regulated gene expression and immune cell infiltration

The immune-related gene dataset was obtained on Genecard and intersected with 145 genes(Supplementary Table S3), resulting in 10 up-regulated immune-related genes (GCG, HLA-DRB5, UCN3, EDN2, PI3, SST, MAPT, BMP3, CMA1, LCN6) and 9 down-regulated immune-related genes (WFIKKN1, QRFP, TAFA1, TRHR, IL13, MIA, INSL4, PLA2G2A, IL9) (Fig. 5A-B, Supplementary Table S4). The Gene Set Enrichment Analysis (GSEA) cellular immunity database was used to evaluate the level of immune cell infiltration in each sample according to the gene expression value in the data set, and the Wilcoxon test was used to test the difference in immune cell infiltration between cluster 1 and cluster 2. The results showed significant differences in the infiltration abundance of activated CD4 T cells, CD56dim natural killer cells, activated CD8 T cells, memory B cells, and Type 2 T helper cells in the two clusters of patients(*P* < 0.05) (Fig. 5C). Further analysis revealed that the expression of immune-related differential genes in STAD patients showed a strong positive correlation with the expression of macrophage cells and Mast cells (Fig. 5D-E).

### Construction of histopathologic DL-signature and gene-signature

**Fig. 6:**
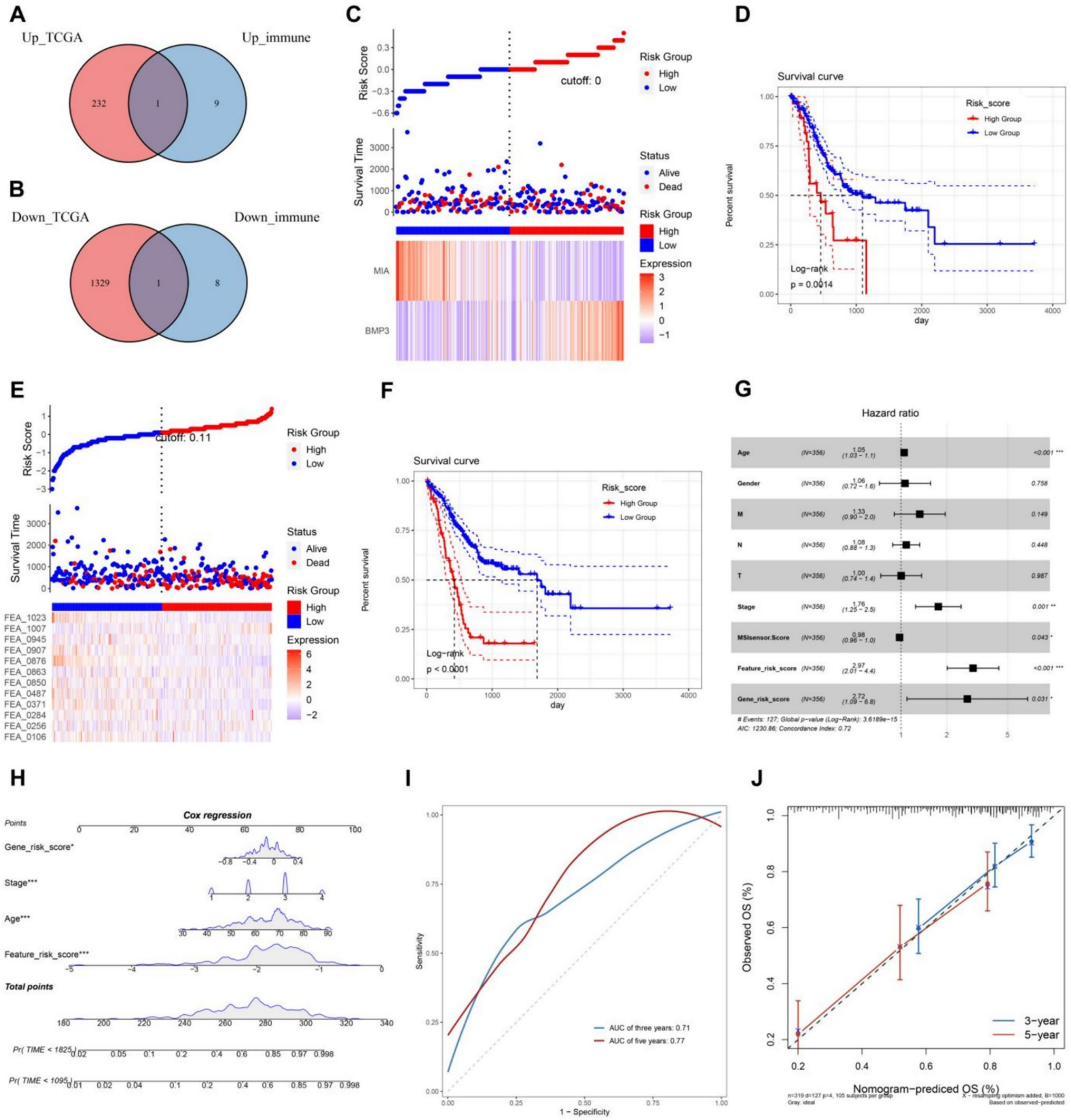
Construction of histopathologic DL-signature and gene-signature. **A** Venn diagram of intersection of up-regulated DEGs and up-regulated immune genes in TCGA-STAD cohort. **B** Venn diagram of intersection of down-regulated DEGs and down-regulated immune genes in TCGA-STAD cohort. **C** The risk heatmap of STAD patients in TCGA-STAD cohort. **D** K-M curves of survival probabilities of high and low risk groups based on gene-signature. **E** The risk heatmap of DL-signature in TCGA-STAD cohort. **F** K-M curves of survival probabilities of high and low risk groups based on DL-signature. **G** Forest plots of multivariate Cox regression of histopathological features, genetic features, and clinical variables. **H** Nomogram included DL-signature,gene-signature and clinical features which predict the 3-, and 5-year overall. **I** Time-dependent ROC of nomogram. **J** Calibration curves of the nomogram for the estimation of 3- and 5-year of OS rates

To construct the histopathological gene signature of STAD, we first classified patients in the TCGA-STAD cohort into two subtypes: mucinous adenocarcinoma and adenocarcinoma, and obtained 232 up-regulated genes and 1329 down-regulated genes by differential analysis(*P* < 0.05, |Log2FC|> 1). These genes were intersected with 19 immune-related genes obtained by pathological features analysis, and one up-regulated gene BMP3 and one down-regulated gene MIA were obtained (Fig. 6A-B). Multivariate cox analysis was used to construct gene signatures, and the distribution of risk scores for patients in the TCGA-STAD cohort, survival status, and relative scores of genes were displayed by heat maps (Fig. 6C). K-M survival curve was drawn to evaluate the survival rate of patients in the high and low-risk groups, and the results showed that the survival rate of the high-risk group was significantly lower than that of the low-risk group(*P* < 0.001) (Fig. 6D). Meanwhile, we constructed a DL-signature based on the relative score of 12 DL features in TCGA-STAD cohort (Fig. 6E). The K-M survival curve showed that the high-risk group had a worse prognosis(*P* < 0.001) (Fig. 6F). Both gene and pathological risk scores had a significant effect on survival. In addition, the forest plot also showed that the pathological and gene feature models we constructed were superior to the clinical features compared with the traditional clinical features (Fig. 6G). Multivariate Cox regression showed that pathological features could be used as independent prognostic predictors of STAD. To provide a comprehensive and accurate approach for prognostic prediction, a nomogram was created using the histopathological DL-signature, gene-signature, and clinical variables of patients from the TCGA-STAD cohort (Fig. 6H-J). The nomogram model can predict 3-year and 5-year OS, which improves the practical application value of histopathological-related features.

## Discussion

Digital pathology can provide valuable information for clinical decision-making and help pathologists to classify histopathological images [24, 25]. Importantly, DL applied to histopathological images also showed good performance in predicting tumor prognosis [26, 27].In this study, we trained a DL model to classify STAD histopathology sections, and the results show that the DL model exhibits high performance in identifying histopathological subtypes (mucinous carcinoma, adenocarcinoma). The AUC value of the model reached 0.90, and it had a high accuracy in the identification of adenocarcinoma and mucinous adenocarcinoma, suggesting that the use of the DL algorithm to assist pathologists in determining pathological classification is an effective means. To further confirm the reliability of this line of thought, we evaluated the model in an external validation set. The results showed that the AUC value of the validation cohort was 0.78, indicating that the model had a certain generalization ability, which provided a theoretical basis for the application of the DL model in STAD pathological classification and recognition. Notably, although the results of the validation cohort hold considerable value, there remains a discernible disparity compared to the outcomes of the training cohort. We guess that this divergence may stem from two potential causes. Firstly, the training cohort was in svs format, while the validation cohort was in ndpi format. This discrepancy could have led to performance variations in the model when processing different image formats, as different formats might employ distinct compression algorithms, color spaces, or image qualities, thereby affecting the accuracy of feature extraction. Secondly, the differing image sizes in the training and validation cohort could also be a factor contributing to the performance disparity.

We divided the TCGA-STAD cohort into two subtypes based on DL characteristics: cluster 1 and cluster 2, and the prognosis of the two subtypes was significantly different, indicating that classification validity is beneficial for predicting the clinical importance of genotype in treatment responsiveness. We also found that mucinous adenocarcinoma was mainly concentrated in cluster 2 and had a poor prognosis, which is consistent with previous reports [28].In addition, we found that DEGs were mainly enriched in signaling pathways such as protein digestion and absorption and enteric nervous system development. Proteolytic enzyme activity and participate in protein absorption transporter expression change can affect the integrity of the gastric epithelium and immune response, thereby promoting tumor growth and progression. It is noteworthy that a high degree of infiltration of the immune microenvironment is present in cluster 2. CD4 + /CD8 + T cells have been reported to partially reflect the infiltration of lymphocytes in gastric cancer tissues, predict the response to immunotherapy to a certain extent, and ultimately affect the tumor progression and survival of gastric cancer [29–31]. CD8 + T cells were associated with improved OS in patients with gastric cancer [32], while high infiltration of CD4 + T cells was correlated with worse OS [33].

Increasingly, researchers are recognizing the cellular properties of the tumor microenvironment (TME), particularly those of immune cells. The tumor immune microenvironment (TIME) plays a crucial role in tumor progression, invasion, metastasis, immune evasion, and treatment resistance [34, 35]. The stomach has strong acidic conditions and a unique endocrine system, which makes the TIME of STAD different. Tumors use diverse mechanisms to evade immune surveillance [36]. These mechanisms include enhancing negative immunomodulatory processes and altering antigen presentation. Populations of immune cells, including tumor-associated macrophages, lymphocytes, tumor-associated neutrophils, T cells, and natural killer cells, play key roles in STAD. Therefore, it is essential to enhance the understanding of the TIME, to identify new targets and improve the clinical efficacy of STAD treatment. The immune-related genes and DEGs were intersected to construct gene signature. The gene signature contains two genes (BMP3, MIA), of which BMP3 has been reported to inhibit the proliferation of STAD by regulating the cell cycle [37], while the mechanism of action of MIA in STAD is not clear. Meanwhile, DL signatures were constructed based on DL features. Both the risk score of gene signature and DL signature were able to significantly influence survival, and further results of multifactorial cox regression showed that DL-signature could serve as an independent prognostic factor. Finally, the systematic nomogram combining DL-signature and gene signature provides some reference value for clinical diagnosis.

Although our study made good progress, some limitations should be noted. First, the DL model was trained and validated. However, the size of the validation cohort was small, and larger samples and specific patient cohorts are needed to evaluate the generalizability of the model in clinical diagnosis. Secondly, the model cannot completely replace the diagnosis of pathological classification by pathologists, who usually need to take into account the influence of clinical factors. In addition, although our model combines transcriptomics analysis to enhance the interpretability of DL-based histopathological classification, the content of the analysis can be further deepened, and the prognosis prediction of STAD patients can be further improved by combining radiomics data of patients in the future.

## Conclusions

In summary, we created a DL model based on histopathological predictive typing and demonstrated that the model recognizes pathological typing with high accuracy. This can be helpful to assist pathologists in making clinical diagnosis. In addition, a nomogram was built by combining DL-signature, gene-signature and clinical features, which can be used as a prognostic classifier for clinical decision-making, individual prognosis and treatment.

## Supplementary Information

Below is the link to the electronic supplementary material.

Supplementary file1 (XLSX 174 KB)

## Data Availability

The WISs, clinical characteristics and mRNA sequencing data of STAD patients were obtained from the TCGA database (https://portal.gdc.cancer.gov/). The validation data set used to develop the deep-learning model is not publicly available because of patient privacy and health information.
